# IUC24397-90 Short-term continence outcomes in men over 75 undergoing robotic-assisted radical prostatectomy

**DOI:** 10.1093/oncolo/oyaf248.032

**Published:** 2025-08-29

**Authors:** D Papanikolaou, D Carbin, S Dranova, D Moschonas, J Hicks, M Kusuma, K Patil, C Eden, M Perry, W A Chedid

**Affiliations:** The Stokes Centre for Urology, Royal Surrey County Hospital, Guildford, United Kingdom; The Stokes Centre for Urology, Royal Surrey County Hospital, Guildford, United Kingdom; The Stokes Centre for Urology, Royal Surrey County Hospital, Guildford, United Kingdom; The Stokes Centre for Urology, Royal Surrey County Hospital, Guildford, United Kingdom; The Stokes Centre for Urology, Royal Surrey County Hospital, Guildford, United Kingdom; The Stokes Centre for Urology, Royal Surrey County Hospital, Guildford, United Kingdom; The Stokes Centre for Urology, Royal Surrey County Hospital, Guildford, United Kingdom; The Stokes Centre for Urology, Royal Surrey County Hospital, Guildford, United Kingdom; The Stokes Centre for Urology, Royal Surrey County Hospital, Guildford, United Kingdom; The Stokes Centre for Urology, Royal Surrey County Hospital, Guildford, United Kingdom

## Abstract

**Background:**

As the incidence of prostate cancer rises with increasing life expectancy, more men over the age of 75 are candidates for curative treatment. Robotic-assisted radical prostatectomy (RARP) is commonly used in younger populations, but data on postoperative continence outcomes in older patients remain limited. To evaluate short-term urinary continence outcomes in men aged ≥75 years undergoing RARP at a high-volume UK center.

**Methods:**

A retrospective analysis was performed on 36 men aged 75 or older who underwent RARP between June 2018 and June 2021. Continence was assessed using the ICIQ-SF questionnaire and pad usage at multiple postoperative time points up to 12 months. Continence was defined as the use of zero pads per day.

**Results:**

At 12 months postoperatively, 63.9% of patients were fully continent (zero pads), and 19.4% used one safety pad daily, yielding an overall functional continence rate of 83.3%. Continence improved progressively over time, consistent with the gradual recovery process observed in this age group.

**Conclusions:**

RARP appears to be a viable surgical option for carefully selected men over 75, offering favorable continence outcomes at 1 year postoperatively. Chronological age alone should not be a contraindication for RARP, and geriatric assessment tools may aid in patient selection. Larger, prospective studies are warranted to confirm these findings.

